# Effects of Induced Astigmatism on Foot Placement Strategies when Stepping onto a Raised Surface

**DOI:** 10.1371/journal.pone.0063351

**Published:** 2013-05-22

**Authors:** Louise Johnson, Elvira Supuk, John G. Buckley, David B Elliott

**Affiliations:** 1 School of Health Studies, University of Bradford, Bradford, West Yorkshire, United Kingdom; 2 Bradford School of Optometry and Vision Science, University of Bradford, Bradford, West Yorkshire, United Kingdom; 3 School of Engineering, Design and Technology, University of Bradford, Bradford, West Yorkshire, United Kingdom; Massachusetts Eye & Ear Infirmary, Harvard Medical School, United States of America

## Abstract

**Purpose:**

Large changes in spectacle prescription can increase falls risk in older people. We investigated the effect of induced astigmatism (a common cause of distorted or blurred vision in older people) on locomotor stepping patterns to determine whether the orientation of astigmatic changes could have differential effects on gait safety when negotiating steps and stairs.

**Methods:**

10 older adults (mean age 76.0±6.4 years) walked up to and stepped onto a raised block whilst wearing their spectacle prescription and when blurred with ±3.00D cylinders at axes 45°, 90°, 135° and 180°. Gait measurements included foot placement before the block, toe clearance over the block edge and foot placement on the block.

**Results:**

Induced astigmatism with axes at 90°, providing magnification in the horizontal meridian only, caused no change in stepping pattern. Induced astigmatism with axes at 180° caused foot placement changes in the anterior or posterior direction according to whether magnification was positive or negative in the vertical meridian (block perceived higher or lower respectively). Induced astigmatism with axes oblique at 45° and 135° (causing the block to be perceived as a parallelogram sloping downwards either to the right or left) caused gait changes in the anterior and posterior, vertical and lateral directions. Changes in lateral foot placement appeared to be an attempt to maintain constant foot clearance levels over the block edge by stepping over the perceived ‘lower’ side of the ‘sloping’ block.

**Conclusions:**

Astigmatic changes with oblique axes had the greatest effect on gait. Clinicians, including optometrists, physiotherapists, occupational therapists and nurses should counsel older patients about the effects of astigmatism on gait safety. Furthermore, partial prescribing of astigmatic corrections should be considered to reduce the risk of falling.

## Introduction

Vision is known to play an important role in postural stability [1], safe negotiation of steps and stairs [2] and the avoidance of obstacles in the travel path [3] and visual impairment has been shown to be a significant risk factor for falls [4], [5]. Given that a significant proportion of visual impairment in elderly people is due to refractive error and cataract [6], it has been suggested that falls could be reduced by updating spectacles and performing cataract surgery [7]–[14]. However, randomized controlled trials (RCTs) of the effects of cataract surgery [11]–[13] and optometric interventions [8], [14] on falls rate have not produced the expected reduction. Two studies found no change in falls rate after cataract surgery [11], [13] and although Harwood and colleagues [12] found a reduction in multiple falls (i.e. two or more) after first eye cataract surgery, there was no change in the overall rate. In their multi-intervention RCT, Day and colleagues [8] found no change in falls rate after ophthalmic intervention alone. Finally and quite surprisingly, the RCT of optometric intervention by Cummings and colleagues [14] reported an increased rate of falls in the intervention group. RCTs of optometric interventions appear limited in that the control group are left to their “usual care”, but because it is relatively easy for the control group to obtain the intervention themselves (and because the possible advantages of the intervention are explained to the control group for ethical reasons), it appears that increased optometric services are gained by both the intervention and control groups [8]. However, this does not explain a significantly *increased* rate of falls in the intervention group, which Cummings and colleagues [14] suggested was due to either increased outdoors activity because of the improved vision provided and/or difficulties adapting to new spectacles causing participants to be at increased risk of falls. The latter was supported by quantitative evidence in that 74% of participants who were given a large change in refractive correction fell compared to 53% who were given a smaller change. It has been suggested that the difficulties adapting to large, changes in spectacle correction are due to changes in spectacle magnification [15], [16]. This has been shown to lead to changes in walking patterns/strategies when negotiating a raised surface [15], [16] and to changes in the vestibulo-ocular reflex gain that can make the world appear to ‘swim’ whilst adapting to the updated spectacle prescription [17]. In the present study, we assessed the effect of induced astigmatism on the everyday gait task of walking up to and stepping onto a raised block. Astigmatism is a common refractive error in older people [18]–[20] and is corrected by glasses that have different powers in different meridians. For example, a person with myopia (short-sightedness) and astigmatism may require a −2.00D power along the horizontal meridian and a −3.00D power along the vertical meridian. This type of astigmatism is common in younger people where eyelid tension tends to steepen the vertical meridian of the cornea at the front of the eye [19]. Changes in astigmatism produce different amounts of magnification along two meridians (which may be along different meridians in the two eyes) so that objects look distorted. Symptoms can include the perception of walls, doors and floors sloping [21], [22]. Optometrists suggest that adapting to new spectacles is more difficult for older adults [22] and it is certainly a major concern for elderly patients attending an eye examination [23]. We systematically blurred vision using +/−3.00D cylinders aligned at various axes. We hypothesized that positive and negative vertically induced astigmatism would produce gait changes similar to those observed following myopic (‘short-sightedness’) and hyperopic (‘long sightedness’) shifts in correction [15], [16] due to vertical magnification effects, with horizontally induced astigmatism showing relatively little effect given that the magnification does not change the perceived block height or slope. The effects on gait of participants perceiving a ‘sloping’ block caused by induced oblique astigmatism, which can occur due to cataract-related changes in older people [18], [20], were unclear.

## Methods

The tenets of the Declaration of Helsinki were followed and the study had approval of the University of Bradford Ethics Committee, with written informed consent being obtained from all participants. Ten fit and healthy older people (mean ± SD, age 76.0±6.4 years, height 1.66±0.10 m, weight 77.8±23.1 kg) were recruited from a group of volunteers who attend training clinics for optometry students at the University. Exclusion criteria included recognised risk factors for falls, for example, diabetes, Parkinson’s disease, significant arthritis and hypotension, and medications known to interfere with balance or coordination, polypharmacy, a history of falls and visual impairment (binocular visual acuity worse than 0.2 logMAR, Snellen equivalent 6/9 or 20/30).

A refraction evaluation incorporating a Jackson cross-cylinder assessment of astigmatism was undertaken and this was compared with the power of the participants’ spectacles. All participants had a refractive correction that was within 0.50D of their current spectacles with minimal difference in astigmatic axis (within 10°).

The everyday gait task of walking up to and onto a raised block (henceforth referred to as the adaptive gait task) was assessed under nine trial conditions: a control condition of their habitual refractive correction (determined by focimetry with the sphero-cylindrical correction being placed into trial frames using full aperture lenses), and their habitual correction plus induced binocular astigmatism using full-aperture cylindrical trial case lenses of +3.00 DC×180, +3.00 DC×90, +3.00 DC×45, +3.00 DC×135, −3.00 DC×180, −3.00 DC×90, −3.00 DC×45, −3.00 DC×135. The participant’s own spectacles were avoided because of the adaptive gait differences between multifocal and single vision spectacles [23], [24]. Our previous studies showed that adaptive gait changes were closely related to the dioptric power of spherical lenses [15] and magnification ‘size’ lenses [16], with the highest-powered lenses having the greatest effects on stepping. In this study, to obtain reasonably sized adaptive gait changes for a selection of cylindrical lens positions (without multiple testing sessions), we used relatively high-powered cylindrical lenses of a size corrected during cataract surgery [18]. These levels of induced astigmatism represent the higher levels of cylindrical correction found in about 3–5% of elderly patients [18]. Cylindrical lenses have zero power along their axis, so that power (and magnification) is provided perpendicular to the axis (x). i.e. a +3.00 DC×180 has zero power along 180° (horizontal) and +3.00D along 90° (vertical). The adaptive gait trials occurred immediately after the blur lenses had been added and no attempt was made to allow the participants to adapt to the lenses. Each lens condition was repeated three times. Binocular visual acuity was measured for all lens conditions using a high contrast ETDRS chart at 4 m with a chart luminance of 160 cd/m^2^, using a by-letter scoring system and a termination rule of 4 letters incorrectly called.

**Figure 1:**
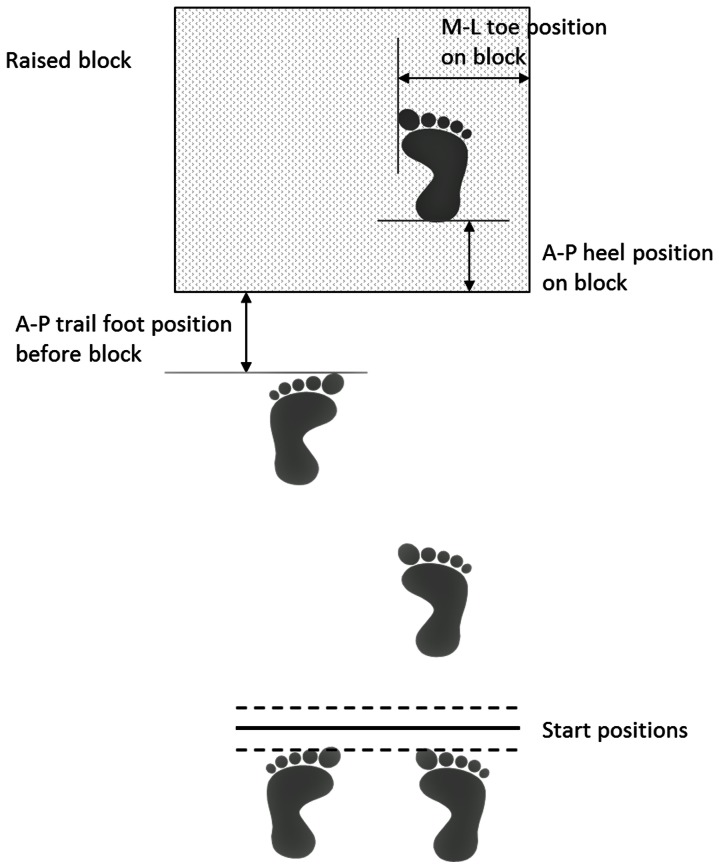
Diagrammatic representation of the gait parameters, including the start positions plus trail foot placement before the block, lead heel antero-posterior placement on the block surface and medial-lateral lead toe placement on the block surface.

The adaptive gait trials consisted of the participant walking up to a 152 mm raised block (width 0.41 m, surface area 0.42×0.46 m) from two walking step lengths away and then stepping onto it and remaining stationary once on the raised block. Two walking steps were chosen as gaze fixation of a step/obstacle in the travel path most frequently occurs at such distances [25]. Once a natural starting position was calculated and marked, additional starting positions of ±5 cm were added. Participants started from one of these three positions, the order of which was randomized [26]. To further discourage the use of a learned motor strategy, eight “dummy trials” were included, where the height of the block was randomly adjusted by −10 mm or +5 mm every fourth trial. No data were collected during these trials and participants were advised that the height of the block would be varied throughout the study [26]. Thus 41 trials (33 trials plus eight dummy trials) were completed. The order of all adaptive gait trials (including the three repetitions) was randomized. The raised block was constructed from medium density fibreboard and was covered in the same green vinyl as the surrounding floor. The laboratory was well lit with ambient illuminance of 400 lux measured at eye level. A member of the research team was positioned near the front edge of the raised block to ensure that if participants should trip or stumble they did not fall. Throughout the experiment participants wore their own, low-heeled or flat-soled shoes, and comfortable clothing. Participants began each trial leading with the right foot and their lead foot that landed on the raised block was their right foot (Figure 1).

Three-dimensional lower limb segmental kinematic data of the stepping pattern were collected (at 100 Hz) using an eight-camera, motion capture system (Vicon MX; Oxford Metrics Ltd, Oxford, UK). Reflective markers (6 mm on feet, 14 mm diameter on other locations) were attached bilaterally either directly onto the clothing or shoes in the following locations: superior aspects of the 2^nd^ and 5^th^ metatarsal heads, end of 2^nd^ toes, lateral malleoli and posterior aspect of the calcanei. Virtual markers, representing the inferior tip of each shoe (virtual shoe tip) were created by determining their locations relative to the markers placed on the 2^nd^ and 5^th^ metatarsal heads and end of 2^nd^ toe. The 3D coordinates of each foot marker (including each virtual shoe tip), and the markers placed on the raised block were exported in ASCII format for further analysis. More details regarding the measurement of the stepping parameters analysed can be found in earlier reports [15], [23], [24]. The toe or heel hitting the step or any momentary loss of balance (typically indicated by the use of the arms to correct balance) when stepping onto the raised block were recorded if agreed by two observers. These trials were included in the mean data reported.

Variables were analysed using repeated measures ANOVA and Tukey’s HSD post-hoc analyses. The statistical package, Statistica 5.5 for Windows (StatSoft, Tulsa, OK) was used and a significant p-value was set at <0.05.

## Results

The mean ±1 SD visual acuity with habitual spectacle correction was 0.06±0.11 logMAR (Snellen equivalent 20/20^−3^). The mean visual acuities with induced astigmatism are given in Table 1. There was a significant main effect of visual acuity for the lens types (F_8,72_ = 19.6, p<0.0001), which was due to better visual acuity with the habitual correction (with no additional lens, i.e., ‘plano’ in Table 1) compared to the other conditions (visual acuity of about 0.35 logMAR, Snellen 20/43) which post-hoc analyses indicated had similar effects (p>0.41).

**Table 1:** Mean ±1 SD visual acuity levels (VA in logMAR) and step negotiation parameters (mm) for 10 older participants with habitual refractive correction and with dioptric blur and magnification changes caused by ±3.00DC with axes at 45°, 90°, 135° and 180°.

	Plano	+3.00 DC×90	−3.00 DC×90	+3.00 180	− 3.00DC×180	+3.00DC×45	− 3.00DC×45	+3.00DC×135	−3.00DC×135
VA	−0.06	0.32	0.31	0.38	0.36	0.41	0.31	0.36	0.34
	0.11	0.12	0.12	0.12	0.14	0.12	0.09	0.10	0.10
Trail	143.7	144.2	147.1	189.8	126.8	177.2	126.6	159.3	150.0
	37.2	40.4	36.0	34.7	44.3	34.1	32.9	38.6	45.8
VTC	44.2	51.8	47.6	61.4	42.4	62.2	40.8	62.3	42.3
	10.0	10.1	5.6	11.6	11.3	13.7	8.9	10.1	11.8
A-P Heel	−48.9	−44.5	−29.9	14.1	−65.5	−14.4	−53.1	−2.7	−52.2
	22.9	24.6	23.0	16.5	40.9	22.4	27.3	22.3	31.0
M-L toe	117.8	116.2	127.2	121.8	117.4	159.3	97.0	91.9	151.7
	16.4	26.0	14.8	13.0	19.9	18.8	18.9	23.5	18.4

Key: Trail foot antero-posterior position before the block (Trail), lead vertical toe clearance (VTC), lead heel antero-posterior position on the block (A–P heel) and medial-lateral position of the toe on the block (M–L toe).

**Figure 2:**
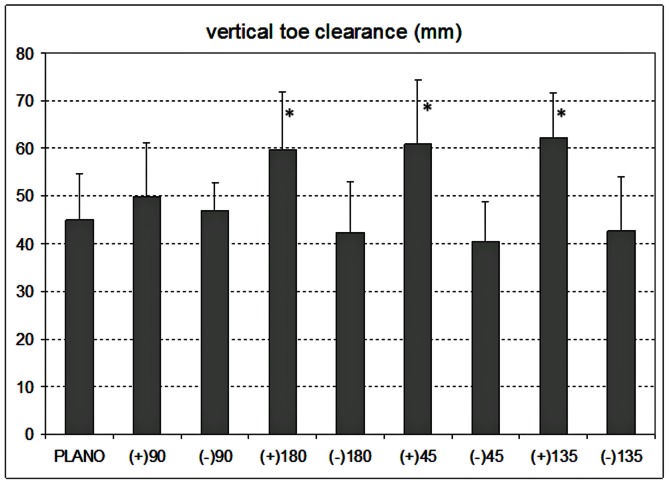
Mean ±1 standard deviation data for lead vertical toe clearance (mm) for 10 older participants with habitual refractive correction plus additional lenses of plano., +3.00 DC×90, −3.00 DC×90, +3.00 DC×180, −3.00 DC×180, +3.00 DC×45, −3.00 DC×45, +3.00 DC×135 and −3.00 DC×135. The top edge of the raised surface is represented at zero mm (y axis). Positive y-axis values correspond to the lead foot being higher than the raised surface. Lens conditions that were significantly different to plano condition are shown by asterisks (p<0.001).

**Figure 3:**
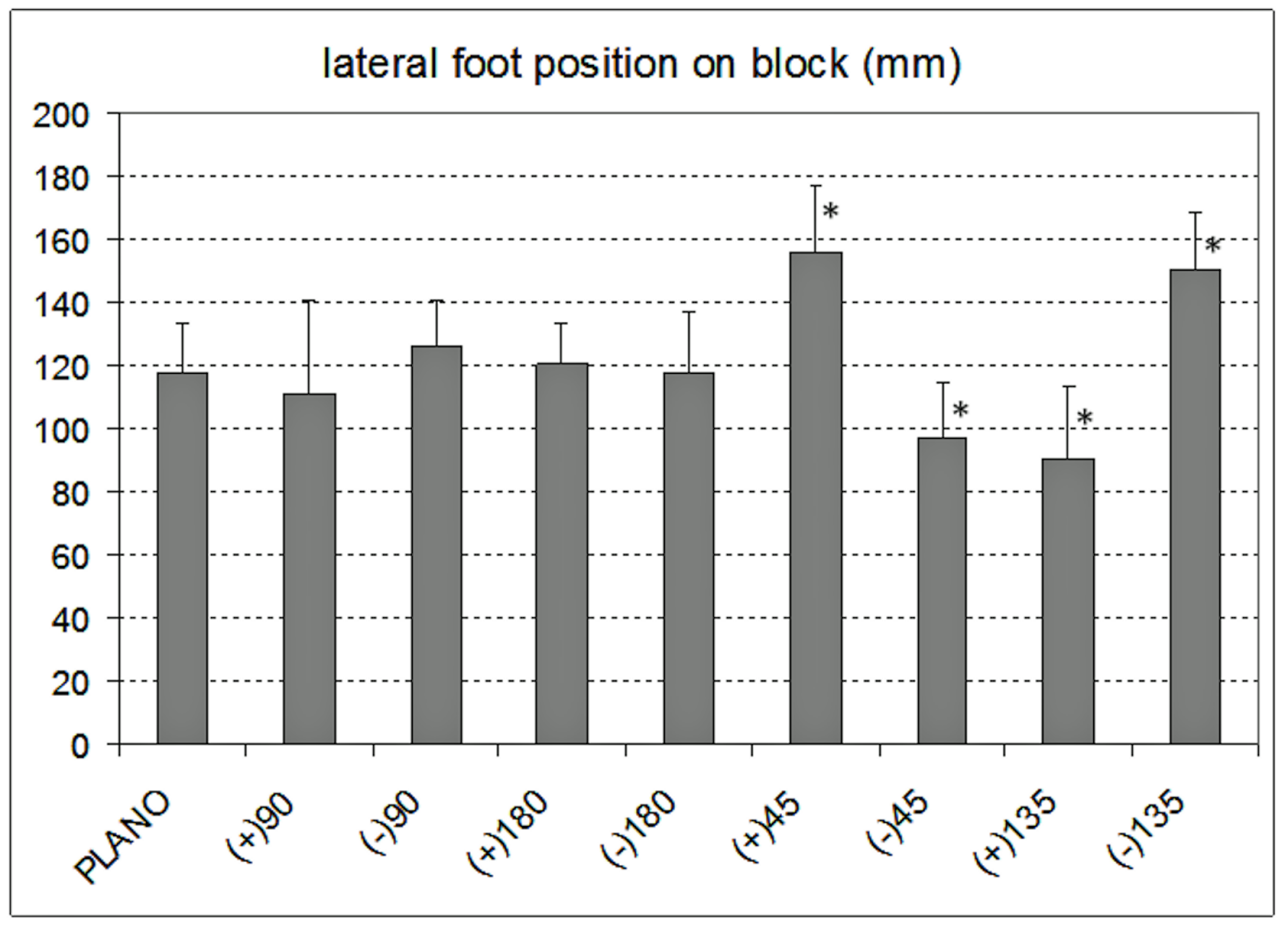
Mean ±1 standard deviation data for medial-lateral lead toe placement on the block surface (mm) for 10 older participants with habitual refractive correction plus additional lenses of plano., +3.00 DC×90, −3.00 DC×90, +3.00 DC×180, −3.00 DC×180, +3.00 DC×45, −3.00 DC×45, +3.00 DC×135 and −3.00 DC×135. The right edge of the block surface is represented at zero mm (width 410 mm; see figure 1). Lens conditions that were significantly different to plano condition are shown by asterisks (p<0.001).

The mean ±1 SD gait parameters for the ten participants are shown in Table 1. Figure 1 depicts how each parameter was defined. Several parameters were significantly affected by the induced astigmatism: trail foot placement before the block (F_8,72_ = 15.2, p<0.0001), lead vertical toe clearance over the block (F_8,72_ = 14.4, p<0.0001; see Figure 2), lead heel antero-posterior placement on the block (F_8,72_ = 28.3, p<0.0001) and medial-lateral lead toe placement on the block (F_8,72_ = 23.9, p<0.0001; see Figure 3). There was a significant repetition or trial effect (F_2,18_ = 15.5, p = 0.0006) only for lead vertical toe clearance, with mean ±1 SD values of 57±19, 50±15 and 44±13 mm for the first, second and third trial. Other parameters showed no significant repetition effect (p>0.19). Post-hoc analyses indicated that all the adaptive gait parameters were similar with plano lenses and astigmatic lenses of +3.00 DC×90 or −3.00 DC×90 (all p>0.13).

Trail foot placement (p<0.001, except +3.00×135, p = 0.59) and vertical toe clearance (all p<0.001) were significantly increased and lead heel antero-posterior placement significantly decreased (i.e. either closer to the block’s front edge or overhanging it, all p<0.0004) with +3.00 DC with axes at 45°, 135° and 180° compared to when wearing plano lenses. Although mean values of trail foot placement and vertical toe clearance were decreased and lead heel antero-posterior placement on the block surface increased with −3.00 DC at 45°, 135° and 180° the values were not significantly different from when wearing plano lenses (p>0.41).

The lead (right) toe medial-lateral placement on the block surface was significantly more to the left (closer to the middle) of the block surface with +3.00 DC×45° (p = 0.0001) and −3.00 DC×135° (p = 0.0002) and was placed significantly more to the right and thus closer to the right hand surface edge of the block with −3.00 DC×45 (p = 0.033) and +3.00 DC×135 (p = 0.0013). There was no change in lead toe medial-lateral placement with plano lenses compared to with ±3.00 DC with axes at 90° and 180° (figure 3; p>0.90). Medial-lateral trail foot placement before the block was affected in a similar manner as lead toe medial-lateral placement (F_8,72_ = 6.8, p<0.0001).

In 16 of 270 trials (5.9% of all trials, which occurred in 7 participants), the toe or heel hit the step or they suffered a momentary loss of balance. This occurred most frequently with additional ±3.00 DC×135 (the only error for three participants; in one participant this led to them slipping off the step to the side due to a large lateral movement) and never occurred under the habitual refractive correction condition.

## Discussion

Visual acuity levels were similar with the addition of all astigmatic lenses, suggesting that the effects on adaptive gait were not due to blur. The lack of changes in gait with positive and negative cylindrical lenses at axes of 90° confirmed our hypothesis of no effect of horizontally induced magnification. This included no change in foot position in the lateral direction. The changes in gait when the cylindrical lenses were oriented with axes at 180° also confirmed our hypothesis that they would produce similar changes to spherical myopic (near-sighted) and hyperopic (long-sighted) shifts in correction [15], [16] due to vertical magnification effects. Compared to the habitual correction with no additional lenses (the ‘plano’ condition) and with cylindrical lenses with axes at 90°, the +3.00DC axis 180° lens resulted in the trail foot being placed further away from the block (p<0.0001), an increased vertical toe clearance as the lead foot crossed the block edge (p<0.0001), and caused the heel to land closer to the leading edge of the block or to land with the heel over-hanging (p<0.0001; see Figure 2). These changes were presumably caused by magnification in the vertical meridian causing the block to look closer and taller than it actually was. This suggests that vertical magnification was driving the changes in gait reported in previous studies with additional spherical lenses [15] and size lenses [16]. The −3.00DC axis 180° lens caused the trail foot to be positioned closer to the block, a decreased vertical toe clearance as the lead foot crossed the block edge, and caused the heel to land further onto the block surface (see Figure 2); although these changes did not reach statistical significance. This would suggest that the study was slightly underpowered to detect these changes (which were not a focus of this study) and that larger changes in gait occurred with the obliquely placed astigmatic lenses. It is possible that the antero-posterior gait changes are determined by the final foot position immediately prior to the block as this will drive the position of the foot as it swings over the block edge before landing on the block [27]. However, as well as gait adaptations being driven by the perceived antero-posterior location of the block it has previously been shown that changing the perceived height of a raised block alone can affect foot clearance over it [28]. It is therefore likely that gait changes are caused by perceived changes in both size and location of the raised block. This highlights the strong link reported between perceptions and actions in adaptive gait [28].

When the cylindrical lenses were placed at oblique axes, magnification was provided along an oblique meridian, so that the rectangular block was perceived more like a sloping parallelogram. The positive cylinders at axes 45° and 135° provided similar antero-posterior gait changes (trail foot position before the block, vertical toe clearance of the lead foot and heel position on landing) to the positive cylindrical lenses at axis 180°, and the negative cylinders at axes 45° and 135° provided similar antero-posterior gait changes to the negative cylindrical lenses at axis 180°, suggesting that these antero-posterior changes were due to the vertical magnification effects of these lenses. However, in addition, these lenses caused lateral changes in position of the trail foot before the block and where the lead foot landed on the block. The +3.00DC axis 45° and −3.00DC axis 135°, which would cause magnification (+3.00DC axis 45°) or minification (−3.00DC axis 135°) in an oblique meridian making the block appear sloped down towards the left (looking from the participant’s viewpoint at the block), caused the lead foot to be placed to the left compared to its position with the non-oblique cylindrical lenses. The −3.00DC axis 45° (minifying) and +3.00DC axis 135° (magnifying), which would make the block appear sloped down towards the right, caused the lead foot to be placed to the right (figure 3). These changes are consistent with the foot being placed towards the lower part of the perceived ‘sloping’ block. The lack of any change in gait with the cylindrical lenses at axis 90° suggests that these changes in lateral foot placements were not due to magnification in the horizontal meridian. These results highlight that maintaining foot clearance at a relatively small level is a prominent driver of adaptive gait, presumably as an energy conservation strategy [29]. The importance of maintaining foot clearance at relatively small levels is also shown by the significant effect of repetition leading to reduced lead toe clearance (from repetition 1 to 2 to 3) [15], [16].

Limitations of the study include the use of high-powered cylindrical lenses that are found in only about 3–5% of elderly patients [18], and it would be useful to repeat the study with lower-powered lenses, although studies using a range of spherical lens [15] and size lens [16] powers suggests that the effects will be similar but slightly smaller.

The results indicate very different effects on adaptive gait of induced astigmatism at different axes. Older adults, who tend to adapt less well to new spectacles [22], [23], should therefore be appropriately warned of potential adaptation problems when prescribed astigmatic change in new spectacles. Astigmatic changes that alter power in the vertical meridian will produce vertical magnification changes and effect gait adaptations in the antero-posterior direction. Oblique astigmatic changes in one or both eyes will make objects such as steps and stairs ‘slope’ and large astigmatic changes can take several days or weeks to adapt to [22]. Similarly, horizontal astigmatic changes in one eye can make objects slope [21]. It would seem sensible that adaptation to new spectacles could be aided by wearing them initially in the home, where surroundings are familiar and the size of steps and stairs are well known, so that perceptual magnification and distortion effects are likely to have less of an effect.

In summary, we investigated the effect of induced astigmatism on stepping patterns to determine whether the orientation of astigmatic changes could have differential effects on gait safety when walking up to and stepping onto a raised block. We found that astigmatic changes with oblique axes have the greatest effect on gait and suggest that older patients in particular should be counseled about the effects of such changes, and partial prescribing of such corrections should be strongly considered [22], [30], particularly given that large changes in refractive correction appear to increase falls risk in older, frail adults [14]. It would also be useful to determine whether full correction of large amounts of astigmatism during cataract surgery [18] has any effect on gait safety and falls.
